# In the Name of COVID-19: Is the ECB Fuelling the Climate Crisis?

**DOI:** 10.1007/s10640-020-00450-z

**Published:** 2020-07-09

**Authors:** T. F. Cojoianu, E. Collins, A. G. F. Hoepner, D. Magill, T. O’Neill, F. I. Schneider

**Affiliations:** 1 Michael Smurfit Graduate Business School and UCD Lochlann Quinn School of Business, University College Dublin, Carysfort Avenue, Blackrock, Co., Dublin, Ireland; 2 School of Geography and the Environment, Oxford University, South Parks Road, Oxford, OX 1 3QY UK; 3 Queen’s University Belfast, Riddel Hall, 185 Stranmillis Road, Belfast, BT9 5EE UK; 4 InfluenceMap, 40 Bermondsey Street, London, SE1 3UD UK; 5 European Commission Technical Expert Group on Sustainable Finance, Brussels, Belgium; 6 Stockholm School of Economics, Mistra Financial Systems (MFS), Stockholm, Sweden

**Keywords:** Green economic recovery, Climate finance, Fossil fuels, Green central banking, Q50, Q58

## Abstract

We offer preliminary evidence drawing on a novel dataset of corporate bonds issued in the European energy sector since January 2020 in combination with the European Central Bank’s (ECB) purchases under the Pandemic Emergency Purchase Programme (PEPP) in response to COVID-19. We show that the likelihood of a European energy company bond to be bought as part of the ECB’s programme increases with the greenhouse gas (GHG) intensity of the bond issuing firm. We also find weaker evidence that the ECB’s PEPP portfolio during the pandemic is likely to become tilted towards companies with anti-climate lobbying activities and companies with less transparent GHG emissions disclosure. Our findings imply that, at later stages of the COVID-19 recovery, an in-depth analysis may be necessary to understand if, and if yes why, the ECB fuelled the climate crisis.

**Electronic supplementary material:**

The online version of this article (10.1007/s10640-020-00450-z) contains supplementary material, which is available to authorized users.

## Introduction

The current global greenhouse gas (GHG) emissions trajectory indicates that the world is likely to experience catastrophic consequences due to climate change, unless swift action is taken towards funding green solutions and the defunding of fossil fuel activities (IPCC 2018). While there is an extensive academic literature on the links between environmental and fiscal policies and the low carbon energy transition (Aghion et al. 2016; Ambec et al. 2013; Cojoianu et al. 2020), as well as on optimal environmental policies in times of economic downturns (van den Bijgaart and Smulders 2018), we know less about what the role of central banks is in promoting a green economic recovery and how monetary policy objectives interact with climate change mitigation objectives in the short and long term (Battiston and Monasterolo 2019; Matikainen et al. 2017).

Given the ambition of the European Union to become a net zero carbon economy by 2050 (Hoepner et al. 2019; Slevin et al. 2020) and the numerous calls to avoid the bailout and stimulus packages towards fossil fuel companies (Hepburn et al. 2020), we examine whether the features of the European Central Bank’s (ECB) €1350 billion Pandemic Emergency Purchase Programme (PEPP) encourages the resilience of the incumbent fossil fuel sector, or whether it promotes the growth of the emerging low carbon energy sector during the COVID-19 pandemic and beyond.

We draw on a novel dataset of corporate bonds issued in the European energy sector between 1st January 2020 and 19th June 2020 in combination with the ECB’s purchases under the PEPP in response to COVID-19. We show that the likelihood of an energy company bond to be bought as part of the ECB’s programme increases with the GHG intensity of the bond issuing firm. We also find weaker evidence that the ECB’s PEPP portfolio during the pandemic is likely to become tilted towards companies with anti-climate lobbying activities and companies with less transparent GHG emissions disclosure in the event of increased Euro-denominated bond issuances in the following months, or re-denominations of non-Euro bonds already issued by European energy companies.

## Policy Background: Climate Change and the ECB

Many central banks remain of the view that central bank interventions should be market-neutral and not discriminate between sectors in the low carbon energy transition (Matikainen et al. 2017). That does not mean, however, that the aim of central banks to remain sector neutral is achievable in practice, as the implementation of the ECB’s post-2008 quantitative easing shows that assets purchased by central banks to stimulate overall economic growth are benefitting more from the policy than assets which are not purchased by the bank (Haldane et al. 2016; Matikainen et al. 2017). This means that the choice of asset class through which asset purchasing programs are implemented matters. This is particularly important in the low carbon economy context, as the fossil fuel energy sector is largely financed through bonds and syndicated bank loans (Cojoianu et al. 2019), whereas much of the emerging clean technology companies are financed through private equity, equity issuances and asset financing (Cojoianu et al. 2020; Gaddy et al. 2017).

Given that the ECB has chosen to enact its asset purchasing program post-2008 crisis predominantly through bonds, this has been shown to favour the incumbent fossil fuel industry (Battiston and Monasterolo 2019; Matikainen et al. 2017), as 62% of ECB’s corporate bond purchases (out of a total of €82 billion) are in GHG intensive sectors—though they make up only 18% of the Eurozone area economy and produce 59% of GHG emissions.

The criteria for the corporate bonds bought under the PEPP are that: (1) the company must be incorporated in the Eurozone and its bond issuance denominated in Euro, (2) the firm cannot be a financial corporation (or a credit institution supervised by the ECB), (3) it cannot be a public entity, (4) the bond issuance has to be endorsed by one positive credit rating by an external credit assessment institution accepted within the Eurosystem credit assessment framework and (5) have a maximum maturity of up to 31 years, and a minimum maturity of 6 months.

## Data and Methodology

In order to understand whether the ECB’s bond buying activity during the COVID-19 pandemic has been tilted towards less transparent, more fossil fuel intensive as well as anti-climate lobbying European energy companies, we undertake the following steps. First, we collect all the bonds issued by European energy companies during the period 1st January 2020 to 19th of June 2020 from Bloomberg. These span the following energy subsectors as classified by BICS (Bloomberg Industry Classification System): power generation, renewable energy, integrated oil and gas companies, oil and gas exploration and production, oil and gas services and utilities. This results in 159 bonds. We then match each bond with the ECB’s bondholding portfolio,^1^ the borrower’s record on pro/anti-climate lobbying from InfluenceMap, the GHG intensity of the borrower (collected from Bloomberg and measured as thousands tCO2-e/million EUR revenue) and the GHG reporting completeness of the borrower (which is assessed by Bloomberg and quantified as 1 if the company is transparent about the organisational boundary it chooses to quantify its GHG emissions and 0 otherwise, Bloomberg terminal code ES074). We further collect the borrower’s revenue (million EUR), bond amount issued (million EUR) and coupon rate for each bond, also from Bloomberg. Our resulting dataset with complete data across all variables of interest is comprised of a cross-section of 68 bonds issued across several currencies, and 52 Euro-denominated bonds.

Our *dependent variable* quantifies the likelihood that the bond of a European energy company is bought by the ECB during the first five months of 2020 and coded as 1 if it has been bought by the ECB, and 0 if it has not. For our model, we employ a binary logistic regression model with robust standard errors. The full model specification is the following, where ε_i_ is the stochastic error:

$$\begin{aligned} ECB Bond_{i} & = \beta_{0} + \beta_{1} *{\text{Pro}} - {\text{Climate Lobbying Activities Score}}_{i} \\ & \quad + \beta_{2} *GHG Emissions Intensity_{i} \\ & \quad + \beta_{3} *GHG Reporting Completeness_{i} \\ & \quad + \beta_{4} *Borrower Revenue_{i} + \beta_{5} *Bond Issuance Amount_{i} \\ & \quad + \beta_{6} *Bond Coupon Rate_{i} + \varepsilon_{i} \\ \end{aligned}$$

## Results and Discussion

We show that after controlling for the revenue of the issuer, the bond amount raised and the rate of the coupon, the ECB is statistically significantly more likely to buy the bonds of more GHG intensive European energy companies (Models 1–4, Table 1). On average, a one standard deviation increase in the GHG intensity of an energy company results in a 147% increase in the likelihood that its bonds are bought by the ECB (β = 0.907, *p* < 0.01, odds ratio: 2.47, Model 3).

**Table 1:** Main statistical models. Likelihood of bond issuance to be bought by ECB. Data from Bloomberg, ECB & InfluenceMap (Logit model)

Dependent variable: ECB = 1 (if bond is purchased by ECB) ECB = 0 (otherwise)	Model 1	Model 2	Model 3	Model 4	Marginal Effects (at mean)	Shapley pseudo R-squared decomposition by factor
	Bond Denomination EUR	Bond Denomination EUR	Bond Denomination EUR	Bond Denomination All currencies	Bond Denomination EUR	
Pro-climate lobbying activities score			− 0.475	− 0.935**	− 0.101	10.92%
			(0.388)	(0.455)	(0.082)	
GHG disclosure completeness		− 0.706	− 0.820	− 1.616***	− 0.175	1.97%
		(0.857)	(0.919)	(0.601)	(0.188)	
GHG intensity	0.983***	1.067***	0.907***	0.983***	0.193***	51.86%
	(0.281)	(0.311)	(0.292)	(0.294)	(0.067)	
Revenue	− 0.608**	− 0.606**	− 0.750***	− 0.702***	− 0.160***	15.05%
	(0.244)	(0.246)	(0.264)	(0.204)	(0.053)	
Bond issuance amount	0.428	0.519	0.217	− 0.217	0.046	3.21%
	(0.584)	(0.647)	(0.700)	(0.413)	(0.148)	
Bond issuance coupon rate	− 0.578	− 0.643	− 0.822	− 1.523***	− 0.175	16.99%
	(0.444)	(0.468)	(0.514)	(0.444)	(0.109)	
Constant	0.379	0.464	0.511	0.236		
	(0.321)	(0.363)	(0.380)	(0.362)		
Observations	52	52	49	68	49	49
Pseudo R-squared	0.163	0.169	0.177	0.348	0.177	0.177 (100%)
Log-likelihood	− 28.56	− 28.36	− 26.04	− 30.66	− 26.04	− 26.04

All variables are standardised (mean = 0 and standard deviation = 1), with the exception of GHG Disclosure Completeness, which takes the value 1 if Scope 1 GHG emissions reporting is transparently reported and 0 otherwise (based on the ES074 score compiled by Bloomberg). Hence the coefficients can be interpreted as a one standard deviation change in the independent variable is related to a β change in the log odds ratio (or e^β^ change in the odds ratio) of the dependent variable. The marginal effects show the coefficient at a one standard deviation increase around the mean of the specific independent variable (as variables are standardised). The Shapley R-squared decomposition shows the relative statistical explanatory power of each independent variable

Significance levels: *p* < 0.01***, *p* < 0.05**, *p* < 0.1*

When we consider only Euro denominated bonds (Models 2 and 3), which are directly under the remit of the ECB, GHG disclosure completeness and pro-climate lobbying are statistically insignificant, yet negative, which suggests that the ECB may be likely to tilt its portfolio towards companies with poorer GHG emission disclosures and less responsible climate lobbying activities. Subsequently, we include the bonds issued by European energy companies in denominations other than Euro, to account for potential sample selection bias due to the choice of energy companies to abstain from issuing Euro denominated bonds as they may have received discouraging signals from the ECB. In other words, analysing the bond issuance of European energy companies in all currencies considers signals that the ECB may have given to the energy companies prior to issuance, while analysing only Euro denominated bonds only considers the observable decision of the ECB to purchase the bonds of specific energy companies post issuance. When we do so (Model 4), it emerges that when considering the entire universe of bonds issued by European energy companies, the ECB’s portfolio is tilted not only to those energy companies that are more GHG intensive, but also to companies which are less transparent on their GHG performance as well as those companies who are more likely to oppose progressive climate action. Having established statistical significance, we investigate the economic and statistical relevance (Brooks et al. 2019). Inspecting the economic relevance of the GHG intensity variable in model 3, we find GHG intensity to have the largest marginal effects. In terms of statistical relevance, we find GHG intensity to have by far the largest Shapley R-squared value, contributing more than 50% to the overall explanatory power of model 3. In conclusion, the importance of GHG intensity is underlined by its marginal economic effects and its statistical relevance, as it explains more variation in the dependent variable on its own than all other variables taken together. We also conduct further robustness tests controlling for bond maturity, bond rating and interactions of key variables and find our results to remain statistically significant.^2^

In conclusion, drawing on a novel dataset of corporate bonds issued in the European energy sector since January 2020 and the database of ECB’s purchases under the PEPP in response to COVID-19, we find evidence that the likelihood for a bond to be bought by the ECB increases with the GHG intensity of the bond issuing firm. We also find weaker evidence that the ECB’s PEPP portfolio during the pandemic is likely to become tilted towards companies with anti-climate lobbying activities and companies with less transparent GHG emissions disclosure.

Our findings imply that, at later stages of the COVID-19 recovery, an in-depth analysis may be necessary to understand if, and if yes why, the ECB fuelled the climate crisis. Even if one accepts that fossil fuel companies were eligible for the PEPP, then our preliminary evidence still raises the significant questions why the ECB was more likely to directly finance those fossil fuel firms that are likely more harmful to the planet (i.e. have a higher GHG intensity)?

## Electronic supplementary material

Below is the link to the electronic supplementary material.

Supplementary material 1 (DOCX 73 kb)

## Footnotes

1 https://www.ecb.europa.eu/mopo/implement/omt/html/index.en.html.

2 These robustness tests as well as more descriptive statistics are available in the Online Appendix.

## References

[CR1] Aghion P, Hemous D, Martin R, Van Reenen J, Dechezleprêtre A (2016). Carbon taxes, path dependency and directed technical change: evidence from the auto industry. J Polit Econ.

[CR2] Ambec S, Cohen MA, Elgie S, Lanoie P (2013). The porter hypothesis at 20: can environmental regulation enhance innovation and competitiveness?. Rev Environ Econ Policy.

[CR3] Battiston S, Monasterolo I (2019). How could the ECB’s monetary policy support the sustainable finance transition?.

[CR4] Brooks C, Hoepner AGF, McMillan D, Vivian A, Wese Simen C (2019). Financial data science: the birth of a new financial research paradigm complementing econometrics?. Eur J Finance.

[CR5] Cojoianu T, Ascui F, Clark GL, Hoepner AGF, Wojcik D (2019) Does the fossil fuel divestment movement impact new oil and gas fundraising? Available at SSRN: http://dx.doi.org/10.2139/ssrn.3376183.

[CR6] Cojoianu T, Clark GL, Hoepner AGF, Veneri P, Wojcik D (2020). Entrepreneurs for a low carbon world: how environmental knowledge and policy shape the creation and financing of green start-ups. Res Policy.

[CR7] Gaddy BE, Sivaram V, Jones TB, Wayman L (2017). Venture capital and cleantech: the wrong model for energy innovation. Energy Policy.

[CR8] Haldane A, Roberts-Sklar M, Wieladek T, Young C (2016) QE: the story so far. https://www.bankofengland.co.uk/working-paper/2016/qe-the-story-so-far. Accessed 30 June 2020

[CR9] Hepburn C, O’Callaghan B, Stern N, Stiglitz J, Zenghelis D (2020). Will COVID-19 fiscal recovery packages accelerate or retard progress on climate change?. Oxf Rev Econ Policy.

[CR10] Hoepner AGF, Masoni P, Kramer B, Slevin D, Hoerter S, Ravanel C, Viñes Fiestas H, Lovisolo S, Wilmotte J-Y, Latini P, Fettes N, Kidney S, Dixson-Decleve S, Claquin T, Blasco JL, Kusterer T, Martínez Pérez J, Suttor-Sorel L, Löffler K, Vitorino E, Pfaff N, Brockmann KL, Micilotta F, Coeslier M, Menou V, Aho A, Fabian N, Philipova E, Hartenberger U, Lacroix M, Baumgarts M, Bolli C, Pinto M, Bukowski M, Krimphoff J (2019). ‘TEG Final Report on Climate Benchmarks and Benchmarks’ ESG Disclosure‘.

[CR11] IPCC (2018). An IPCC Special Report on the impacts of global warming of 1.5°C. Intergov Panel Clim Change.

[CR12] Matikainen S, Campiglio E, Zenghelis D (2017) The climate impact of quantitative easing. Policy Paper, Grantham Research Institute on Climate Change and the Environment, London School of Economics and Political Science

[CR13] Slevin D, Hoerter S, Humphreys N, Viñes Fiestas H, Lovisolo S, Wilmotte J-Y, Latini P, Fettes N, Kidney S, Dixson-Decleve S, Claquin T, Blasco JL, Kusterer T, Martínez Pérez J, Philipponnat T, Löffler K, Vitorino E, Pfaff N, Brockmann KL, Redondo Pereira P, Coeslier M, Menou V, Aho A, Fabian N, Hartenberger U, Lacroix M, Baumgarts M, Bolli C, Philipova E, Pinto M, Bukowski M, Krimphoff J, Hoepner AGF, Masoni P, Kramer B (2020). Taxonomy: final report of the Technical Expert Group on Sustainable Finance.

[CR14] van den Bijgaart IM, Smulders S (2018). Does a recession call for less stringent environmental policy? A partial-equilibrium second-best analysis. Environ Resour Econ.

