# Supplementary material for: In the Name of COVID-19: Is the ECB Fuelling the Climate Crisis?
Source: Environ Resour Econ (Dordr). 2020 Jul 9:1–7. Online ahead of print. doi: 10.1007/s10640-020-00450-z (PMC7343577; doi:10.1007/s10640-020-00450-z)
Supplement: Supplementary file 1 — Supplementary material 1 (DOCX 73 kb) [file 10640_2020_450_MOESM1_ESM.docx]

# Online Appendix Only

**Extended descriptive analysis**

The European energy sector fundraised over €94 billion from bond investors during the January to May 2020 period. Over 50% of this amount went to the oil & gas sector, 34% to utilities, 8% to power generation and only 6% to renewable energy companies (see Fig. OA.1). A large proportion of this debt has been issued in April and May 2020, when perhaps companies expected the liquidity gain and the lowering of their credit risk if their bonds are bought by the ECB, who made the PEPP public on 18^th^ of March 2020.

For the oil and gas sector, the largest proportion of its newly issued debt matures between 2030 and 2040 (c. €22 billion), with the 2020 -2030 and beyond 2040 periods accounting for c. €18 billion and €6.5 billion respectively (see Fig. OA.2). This is very important as none of this debt, part of which was bought by the ECB, is contingent on the energy companies reducing their GHG footprint, ceasing their anti- climate lobbying activities or reporting their GHG emissions more accurately.


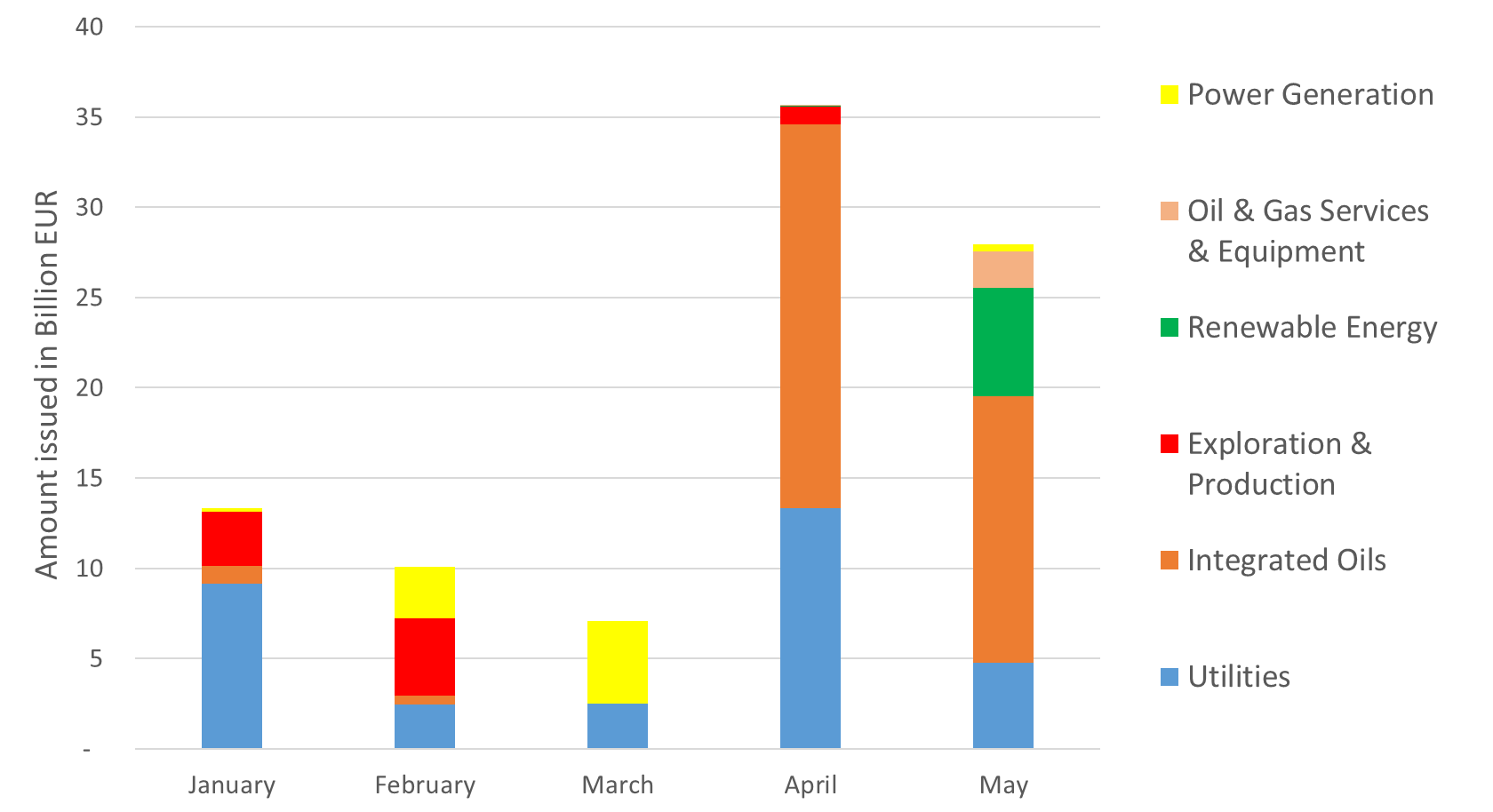


**Figure OA. 1:** Bond issuance by European energy companies Jan – May 2020. Data from Bloomberg. Source: Authors.


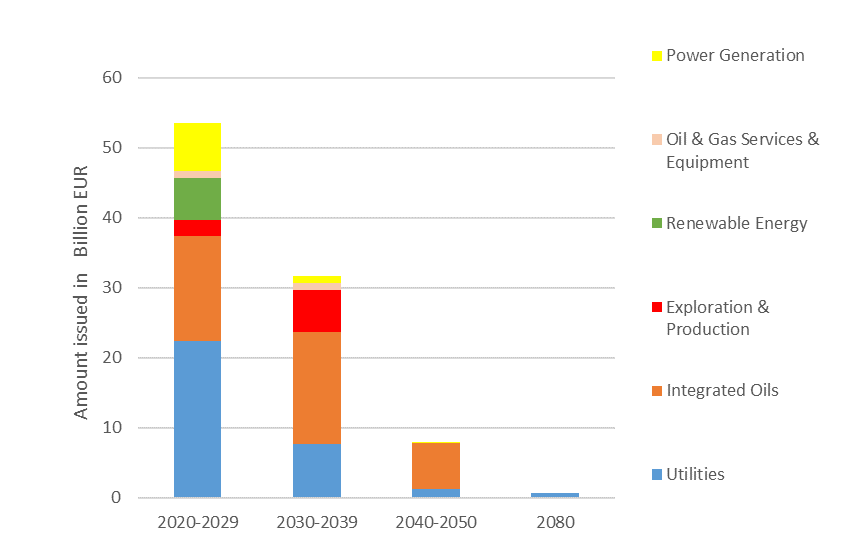


**Figure OA.2:** Maturities of European energy company bonds issued between Jan – May 2020. Data from Bloomberg. Source: Authors

**Further robustness tests**

**Table OA.1:** Further robustness tests – controlling for bond maturity and bond rating.

| Dependent variable: ECB = 1 (if bond is purchased by ECB)  ECB = 0 (otherwise) | Robustness test | Robustness test |
| --- | --- | --- |
|  | Controlling for Bond Rating  (Bond Denomination EUR) | Controlling for Bond Maturity  (Bond Denomination EUR) |
| Pro-Climate Lobbying Activities Score | -1.057 | -0.338 |
|  | (1.145) | (0.379) |
| GHG Disclosure Completeness | -7.012** | -0.822 |
|  | (3.559) | (0.968) |
| GHG Intensity | 1.337** | 1.077*** |
|  | (0.581) | (0.376) |
| Revenue | -1.953*** | -1.020** |
|  | (0.640) | (0.400) |
| Bond Issuance Amount | -0.756 | -0.075 |
|  | (1.259) | (0.717) |
| Bond Issuance Coupon Rate | -0.518 | 0.321 |
|  | (1.216) | (0.833) |
| Bond Maturity |  | -0.453 |
|  |  | (0.403) |
| Bond Rating Control | YES | No |
| Observations | 34 | 47 |
| Pseudo R-squared | 0.263 | 0.208 |
| Log-likelihood | -16.67 | -23.30 |
| Significance levels: p <0.01***, p <0.05**, p<0.1*. All variables are standardized (mean = 0 and standard deviation = 1), with the exception of GHG Disclosure Completeness, which takes the value 1 if Scope 1 GHG emissions reporting is transparently reported and 0 otherwise (based on the ES074 score compiled by Bloomberg). Hence the coefficients can be interpreted as a one standard deviation change in the independent variable is related to a β change in the log odds ratio (or e^β^ change in the odds ratio) of the dependent variable. The marginal effects show the coefficient at a one standard deviation increase around the mean of the specific independent variable (as variables are standardised). | | |

**Table OA.2:** Marginal effects at mean for independent and control variables (logit model)

| Dependent variable: ECB = 1 (if bond is purchased by ECB)  ECB = 0 (otherwise) | Marginal Effects  (at mean) | Marginal Effects  (at mean) |
| --- | --- | --- |
|  | Bond Denomination EUR | Bond Denomination All currencies |
| Pro-Climate Lobbying Activities Score | -0.101 | -0.229** |
|  | (0.082) | (0.111) |
| GHG Disclosure Completeness | -0.175 | -0.396*** |
|  | (0.188) | (0.152) |
| GHG Intensity | 0.193*** | 0.240*** |
|  | (0.067) | (0.071) |
| Revenue | -0.160*** | -0.172*** |
|  | (0.053) | (0.052) |
| Bond Issuance Amount | 0.046 | -0.053 |
|  | (0.148) | (0.101) |
| Bond Issuance Coupon Rate | -0.175 | -0.373*** |
|  | (0.109) | (0.112) |
| Observations | 49 | 68 |
| Pseudo R-squared | 0.177 | 0.348 |
| Log-likelihood | -26.04 | -30.66 |
| Significance levels: p <0.01***, p <0.05**, p<0.1*. All variables are standardised (mean = 0 and standard deviation = 1), with the exception of GHG Disclosure Completeness, which takes the value 1 if Scope 1 GHG emissions reporting is transparently reported and 0 otherwise (based on the ES074 score compiled by Bloomberg). Hence the coefficients can be interpreted as a one standard deviation change in the independent variable is related to a β change in the log odds ratio (or e^β^ change in the odds ratio) of the dependent variable. The marginal effects show the coefficient at a one standard deviation increase around the mean of the specific independent variable (as variables are standardised). | | |

**Table OA.3:** Shapley Pseudo R-squared decomposition by variable

| Factor | Shapley Value | Per cent estimate  (out of total R-squared) |
| --- | --- | --- |
| Pro-Climate Lobbying Activities Score | 0.019 | 10.92% |
| GHG Disclosure Completeness | 0.003 | 1.97% |
| GHG Intensity | 0.091 | 51.86% |
| Revenue | 0.027 | 15.05% |
| Bond Issuance Amount | 0.005 | 3.21% |
| Bond Issuance Coupon Rate | 0.030 | 16.99% |
| Total | 0.177 | 100% |
| Note: Computed using the Shapley2 package in Stata. Shapley values show the average improvement in the explanatory power of the model by including a particular variable. The per cent estimates show the relative importance of each variable in the explanatory power of the model. | | |

**Table OA.4:** GHG intensity – revenue interaction effects robustness test.

| Dependent variable: ECB = 1 (if bond is purchased by ECB)  ECB = 0 (otherwise) | GHG Intensity – Revenue Interaction |
| --- | --- |
|  | Bond Denomination All Currencies |
| Pro-Climate Lobbying Activities Score | -1.012** |
|  | (0.465) |
| GHG Disclosure Completeness | -1.913** |
|  | (0.812) |
| GHG Intensity | 0.768* |
|  | (0.401) |
| Revenue | 0.639* |
|  | (0.350) |
| GHG Intensity x Revenue | 0.001 |
|  | (0.032) |
| Bond Issuance Amount | -0.483 |
|  | (0.349) |
| Bond Issuance Coupon Rate | -1.805*** |
|  | (0.528) |
| Constant | -5.836* |
|  | (3.261) |
| Observations | 68 |
| Pseudo R-squared | 0.368 |
| Log-likelihood | -29.70 |
| Significance levels: p <0.01***, p <0.05**, p<0.1*. Given that GHG intensity is defined as Absolute GHG Emissions / Revenue, interacting it with the Revenue variable results in the Absolute GHG Emissions effect, which in this case is not statistically significant. | |

**Table OA.5:** Correlation matrix.

| Variables | Mean | St. Dev. | Min | | Max | | (1) | | (2) | (3) | (4) | | (5) | | (6) |
| --- | --- | --- | --- | --- | --- | --- | --- | --- | --- | --- | --- | --- | --- | --- | --- |
| (1) Pro-Climate Lobbying Activities Score | 57.7 | 14.7 | | 32.04 | | 89.62 | | 1.000 |  |  |  |  |  |  |  |
| (2) GHG Disclosure Completeness | 0.19 | 0.39 | | 0 | | 1 | | -0.145 | 1.000 |  |  |  |  |  |  |
| (3) GHG Intensity | 20.11 | 29.78 | | 0.001 | | 119.78 | | -0.156 | 0.097 | 1.000 | |  |  |  |  |
| (4) Revenue | 281631 | 1371548 | 0 | | | 7659623 | | -0.261** | -0.088 | -0.149 | | 1.000 | |  |  |
| (5) Bond Issuance Amount | 781 | 435.96 | 5 | | 2500 | | -0.406*** | | 0.244** | 0.261** | | 0.321*** | | 1.000 |  |
| (6) Bond Issuance Coupon Rate | 1.79 | 1.19 | 0 | | 6.75 | | -0.304*** | | 0.160 | -0.051 | 0.263** | | 0.441*** | | 1.000 |

**InfluenceMap Climate Lobbying Score Methodology**

The lobbying scores as used in the model were calculated by InfluenceMap using the following methodology, also outlined on the InfluenceMap website.^[[1]](#footnote-1)^ The top 100 of the Forbes Global 2000 (not including state owned enterprises and financial companies) were selected as well as external influencers identified. ‘Influencers’ are a selection of the most powerful organizations representing corporations around the world. Influence likely extends beyond the activities normally associated with the word "lobbying" (e.g. donations to clearly motivated political actors) and includes the domination of the public discourse on climate change science and policy via their hugely powerful and funded messaging tools (e.g. advertising, PR, social media, access to influential meetings) as well as the use of influencers like trade associations and advocacy groups. Climate-focused organizations were excluded to avoid preferential selection. The process of climate change policy is broken down into four distinct categories as formulation of climate change policy commences with scientific research that then enters the policy arena, leading to the implementation of legislation, standards and fiscal measures. In parallel, many legislative and fiscal interventions are in place or are being proposed at various levels of government. Corporations have been interacting with policymakers at all levels and stages of the policy formulation process, using various methods to exert influence. The queries introduced by InfluenceMap cover all of these stages and are divided into two main categories: Transparency and performance.

In total, a series of twelve queries is applied across all data sources, constructing a matrix of queries against data sources for each organization. While this matrix presents an opportunity for a maximum of 96 - i.e. 12 x 8 - scoring opportunities per organization, in practice this will be less due to the NA (not applicable) and NS (not scored) cells for a particular organization's matrix. Data originating from the last two years is considered while priority is given to more recent evidence.

Relative weightings are assigned to each query/data cell relative to the overall organizational score. A generic weighting matrix is applied to all sectors, yet certain sectors will have a sector specific weighting matrix emphasizing its legislative priorities (e.g. the automotive sector will be weighted more for its influence over GHG emissions standards than energy policy). The weightings are devised from InfluenceMap’s independent research and in consultation with advisors and external experts.

The matrix of data source/query produces five possible outcomes with scores ranging from (-2) to (+2) depending on an organization’s transparency around particular regulations, their expression of support (or non-support), and the corresponding strength of their engagement with this regulation. As well as its organizational score, the final rating for a corporation will be impacted by the relationships (R1, R2, R3, etc.) it holds with external agents exerting influence over climate policy, such as trade associations, chambers of commerce and think tanks. Therefore, in addition to its organizational score, a corporation will have a relationship score which is defined as a reflection back onto the corporation on the influence exerted by its influencers. The influencers will themselves have organizational scores, computed in the same manner as for the corporations. To account for the nature of a corporation’s relationship with an influencer a strength factor is applied to the relationship (1 = a weak relationship, 10 = a strong relationship).

The relative weighting as a metric of the level of influence exerted by the influencer with which the corporation holds a relationship is defined comparing to those of other influencers in the global policy arena. Levels of influence against each other are rated on a scale of 1 to 10 (with 10 being very important as an influencer of climate policy).

The overall rating is obtained by computing the weighted average of the two individual scores, organizational and relationship score. The weighting factor is between 0 and 1 and determined using an algorithm that incorporates both strength and relative weight of the corporation and also the number of relationships. This avoids a small sample of relationships to unduly impact the overall rating for a corporation.

1. <https://influencemap.org/site/data/000/286/Methodology.pdf> [↑](#footnote-ref-1)
